# Extracellular vesicles nanoarray technology: Immobilization of individual extracellular vesicles on nanopatterned polyethylene glycol-lipid conjugate brushes

**DOI:** 10.1371/journal.pone.0224091

**Published:** 2019-10-24

**Authors:** Shusuke Yokota, Hiromi Kuramochi, Kyohei Okubo, Akiko Iwaya, Shoichi Tsuchiya, Takanori Ichiki

**Affiliations:** 1 Department of Materials Engineering, School of Engineering, The University of Tokyo, Bunkyo, Tokyo, Japan; 2 Innovation Center of NanoMedicine, Kawasaki, Kanagawa, Japan; LAAS-CNRS, FRANCE

## Abstract

Arraying individual extracellular vesicles (EVs) on a chip is expected one of the promising approaches for investigating their inherent properties. In this study, we immobilized individual EVs on a surface using a nanopatterned tethering chip-based versatile platform. A microfluidic device was used to ensure soft, reproducible exposure of the EVs over the whole chip surface. The device is incorporated with a high-density nanoarray chip patterned with 200-nm diameter nanospots composed of polyethylene glycol (PEG)-lipid conjugate brushes. We present a procedure adopted for fabricating high-density PEG-lipid modified nanospots (200 nmϕ, 5.0 × 10^5^ spots/mm^2^ in 2 × 2 mm^2^ area). This procedure involves nanopatterning using electron beam lithography, followed by multistep selective chemical modification. Aqueous treatment of a silane coupling agent, used as a linker between PEG-lipid molecules and the silicon surface, was the key step that enabled surface modification using a nanopatterned resist film as a mask. The nanoarray chip was removed from the device for subsequent measurements such as atomic force microscopy (AFM). We developed a prototype device and individually immobilized EVs derived from different cell lines (Sk-Br-3 and HEK293) on tethering nanospots. We characterized EV's morphology using AFM and showed the possibility of evaluating the deformability of EVs using the aspect ratio as an indicator.

## Introduction

Extracellular vesicles (EVs) have attracted considerable attention due to their potential application as diagnostic biomarkers [1–4]. EVs are secreted by most cell types through fusion with cell membranes and circulate in the blood. They can also serve as mediators for intercellular communication by transferring encapsulated proteins, deoxyribonucleic acid (DNA), messenger ribonucleic acid (mRNA), and microRNA (miRNA). Given that exosomal miRNAs have been empirically linked to the initiation and progression of various human cancers, miRNAs (short noncoding RNAs that post-transcriptionally regulate the expression of target genes) are expected to be primary candidates for cancer biomarkers [5]. A number of studies have reported that miRNAs encapsulated in EVs regulate the surrounding cells [6–8], and the circulation of EVs ensures that metastasis is formed at distant tissues [9]. The medical significance of the diversity within the EV population is now extensively recognized [10]. However, at the same time, it is well known that EVs differ in sizes and functions due to their origins and purification methods [11–15] that prevents a clear understanding of their nature and clinical applications. Therefore, it is vital to characterize individual EVs to investigate their inherent heterogeneity.

Thus, it is necessary to develop analytical methods for studying individual EVs. For instance, the following analytical methods have been employed to study a population of EVs: spectrophotometry, mass spectrometry, and Western blotting for quantifying proteins; microarrays for profiling miRNAs; and dynamic light scattering (DLS) for obtaining particle size distribution [16]. Atomic force microscopy (AFM) [17–19] and transmission electron microscopy [20] have been used in individual EV studies to investigate nanoscale structures and size distribution. Through nanoparticle tracking analysis (NTA), on-chip microcapillary electrophoresis enables the profiling of protein expressions [21]. Surface-enhanced Raman spectroscopy (SERS) provides fingerprint spectra of membrane proteins, which helps to distinguish cancer-derived EVs [22, 23]. Moreover, nanofluidic-based flow cytometers fluorescently detect labeled EVs in solutions [24]. It is necessary to further develop high-throughput, non-invasive, and multiplexed analysis methods to proceed with individual EV studies.

High-density arraying of single EVs on a surface is a promising approach for studying EVs using a combination of established analytical techniques [25]. Microarray technologies, such as DNA arrays [26] and protein arrays [27], are obtained by patterning thousands of molecules in an array-like format to perform a high-throughput assay of biological systems. The array platform can be applied to cells to investigate cellular responses at single-cell levels [28]. Similarly, EV arrays are considered powerful and versatile tools for bioassays. When envisioning multiplexed omics analysis and classification of individual EVs, the array method can specify the address of objects, thereby ensuring the possibility of comprehensive EV analysis in one-to-one correspondence with multiple characteristics. Moreover, array methods that enable specific addresses are advantageous for incorporating machine learning algorithm [29] into omics analysis.

In this study, we propose a versatile platform for EV analysis using a tethering nanoarray chip. It uses an array of nanospots comprising polyethylene glycol (PEG)-lipids as anchors for EV capture through hydrophobic interactions [30, 31]. Using hybrid nanofabrication technologies based on electron beam (EB) lithography and selective chemical modification, we designed the nanospots to have sizes comparable to those of the EVs. EVs derived from different cells were immobilized on the tethering nanoarray by exposing the EV suspension onto the chip using a microfluidic device. The immobilized EVs were measured through AFM to compare their deformability. To the best of our knowledge, this is the first study that demonstrates an EV nanoarray platform involving nanopatterned PEG-lipid conjugate brushes.

## Concept and design

**Fig 1:**
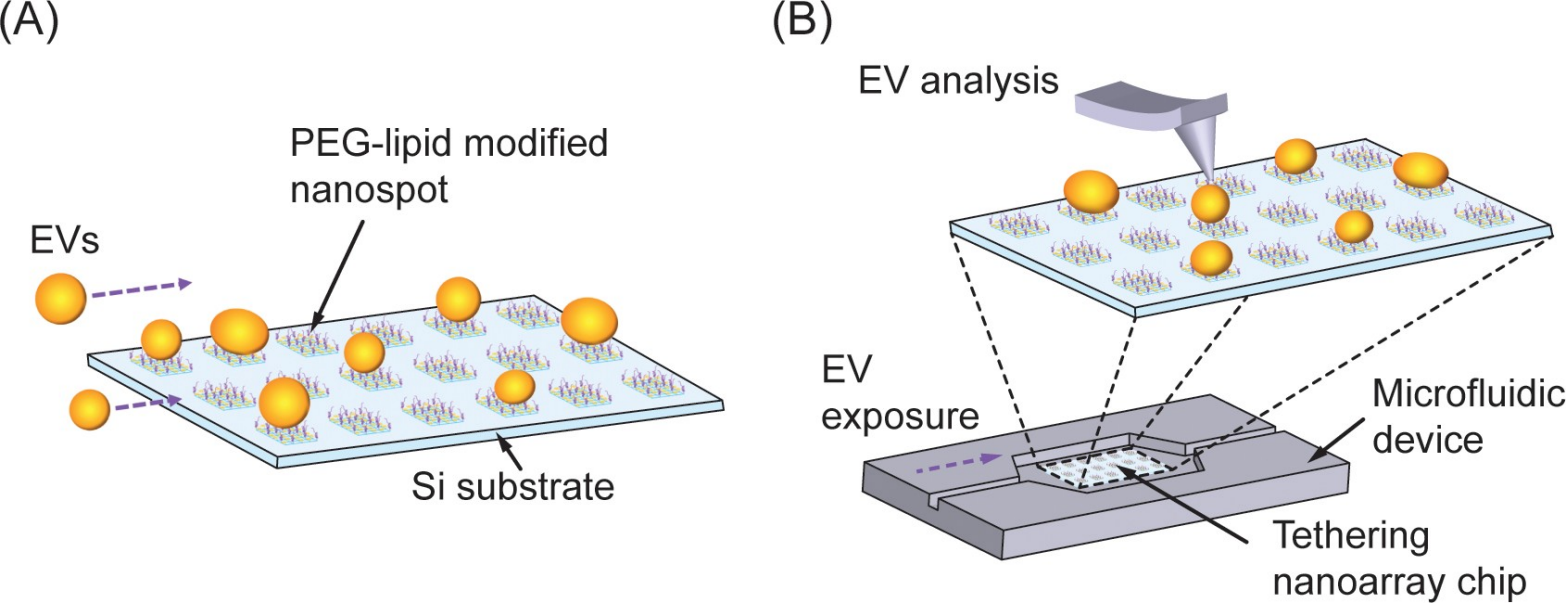
Concept of tethering nanoarray platform for individual EV study. (A) Schematic of tethering nanoarray chip. (B) Concept of analysis platform using a tethering nanoarray chip and a microfluidic device. The nanoarray chip can be detached from the microfluidic device for EV analysis.

To individually immobilize EVs on a surface, it is vital for us to select appropriate capture-probe molecules to obtain a versatile array platform. Using antibodies that specifically bind to membrane molecules ensures that immunoaffinity-based methods can be adopted because EV markers such as CD9, CD63, and CD81 can immobilize EVs on the surface. However, considering the heterogeneity of EVs, using antibodies might cause unexpected bias in the types of EV captured. To capture liposomes and spherical vesicles comprising phospholipid membranes, lipophilic anchors containing alkane chains [32], cholesterol [33], or lipid-oligonucleotide conjugates [34] have been used in previous reports. Therefore, to avoid unexpected bias, we used lipophilic anchors as capture-probe molecules in the platform. They include PEG-lipid conjugate brushes that facilitate the capture of lipid bilayer membranes via hydrophobic interactions between the oleyl groups and the lipid bilayer membranes [35]. As shown in Fig 1A, we designed the tethering array of the PEG-lipid conjugate brushes to have comparable sizes to those of the EVs in order to individually immobilize the EVs. The tethering nanoarray chip was integrated into a microfluidic device to ensure reproducible exposure of EV suspensions over the chip surface. After exposing the sample, it is easy to detach the nanoarray chip from the microfluidic device for further investigation of individual EVs tethered on the chip (Fig 1B).

The diameter of nanospots of the array was selected following a previous work [36] in which a coarse-grain model was used to predict the shape of membrane vesicles adsorbed on a solid surface. The model demonstrated a 100-nm diameter EV in suspension, which was deformed with a diameter of 150 nm when bound to the SiO_2_ surface. We designed an array chip of 200-nm diameter nanospots with a pitch of 200 nm and a density of 5.0 **×** 10^5^ spot/mm^2^. The tethering nanoarray chips were produced using a hybrid approach that utilizes top-down and bottom-up methods such as EB lithography, lift-off process, and selective chemical modification. To fabricate the PEG-lipid modified nanospots, aqueous 3-aminopropyltriethoxysilane (APTES) solution [37, 38] was employed for selective amino-silanization using a nanopatterned resist film as a mask.

## Material and methods

### Fabrication of tethering nanoarray chip

Reagents and materials used for fabricating the chips were obtained from the following commercial sources. Silicon (Si) (100) wafers from Nilaco; positive EB resist (ZEP520A), developer (ZED-N50), and rinse solution (ZMD-B) from Zeon Corp; 3-aminopropyltriethoxysilane (APTES) from Sigma-Aldrich; dimethylacetamide (DMAC) from Wako; PEG-lipids (Mw 8,000, OE-080CS) and Methoxy PEG (Mw 2,000, ME-020AS) from NOF Corp. All the solutions were prepared using distilled-deionized water.

EB lithography was performed using Advantest F7000S-VD03 system. We observed nanopatterned resist films using a scanning electron microscope (SEM, JSM-7500F, JEOL Ltd.). We directly evaluated the sizes of the patterned nanoholes from the SEM images. Details of the chemical modification process and evaluation are provided in supporting information (S1 Text and S1 Fig).

### Design and fabrication of the microfluidic device

The microfluidic device comprises a chamber (variable-size according to the chip; typical size for this experiment: width, 20 mm; length, 20 mm; height, < 500 μm) used for placing the tethering nanoarray chip, sample inlet/outlet ports (diameter, 4 mm), channels (width, 2 mm; depth, 1 mm) to connect the chamber and inlet/outlet holes. The structure of the components was engraved on an 80 **×** 100 mm^2^ polystyrene methacrylate (MS, JSP) plate using a computer numerical control milling machine (MDX-540, Roland DG). The processed plate was ultrasonically flushed in ethanol for 1 min using a supersonic cleaner (Bransonic1510, Branson). Subsequently, it was flushed with deionized water and dried under a stream of dry nitrogen. A homemade suction pump was connected to the outlet hole to control the flow rate of the EV suspensions and rinse solutions [35]. In the microfluidic device, a peristatic pump system based on silicone elastomer valves can be embedded along the channels to allow EV immobilization under circulating flow. This function is shown in S1 Movie.

### Preparation and measurement of EVs

The EVs examined in this study were obtained from human breast cancer Sk-Br-3 cell lines (HTB-30, ATCC) and human embryonic kidney (HEK)293 cell lines (RIKEN BRC).

The Sk-Br-3 cells were cultured to confluence using Dulbecco's Modified Eagle Medium (DMEM, 11885–084, Gibco) supplemented with 10% bovine serum albumin (Fetal Bovine Serum: FBS), 100 units/ml penicillin, 0.1 mg/ml streptomycin, and 0.1 mg/ml kanamycin. To remove the FBS-derived EV, the medium was replaced with serum-free medium and cultured for 48 h. The supernatant was then recovered and the EV sample purified by ultracentrifugation method (300 **×**g, 10 min; 2,000 **×**g, 20 min; 10,000 **×**g, 100 min; 100,000 **×**g, 200 min; 100,000 **×**g, 200 min). We made a density gradient solution using OptiPrep (Alere Technologies AS) and placed the EV suspension on it and centrifuged at 100,000 **×**g for 16 h. After centrifugation, the density fraction was recovered using gradient station (Biocomp). The collected fractions were suspended in phosphate-buffered saline (PBS, pH = 7.4, Thermo Fisher Scientific) and centrifuged at 100,000 **×**g for 200 min to remove the remaining sucrose. The supernatant was discarded, and the pellet was suspended in PBS. A fraction with a density of 1.124 g/ml [39–41] was used for measurements, after examining the content of EV markers in each fraction using Western blotting (see S2 Fig for detailed information).

The HEK293 cells were mixed with DMEM to yield 10% FBS. To prevent contamination by bacteria, the antibiotics penicillin, kanamycin, and streptomycin were mixed to a concentration of 1%, and the cells were cultured to a confluent state. The medium replaced DMEM with antibiotics to remove EVs derived from FBS, and the cells were cultured at 37°C, 5% CO_2_, 95% air for 48 hours. The EV sample was purified by ultracentrifugation (2,000 **×**g, 15 min; 12,000 **×**g 100 min; 110,000 **×**g, 200 min; 110,000 **×**g, 200 min). This step is vital for filtering most lipoproteins from EV suspensions.

We measured the particle size distribution of the collected EVs (see S3 Fig for detailed information) using DLS [42]. The Brownian motion of each EV was visualized using scattered light and recorded for 60 s to calculate its size using the Stokes–Einstein equation.

AFM measurements were performed in intermittent contact (tapping) mode using a JPK NanoWizard III AFM (JPK Instruments AG) in water to observe the fabricated nanospots and in PBS to evaluate the morphology of EVs tethered on the nanospots. For each of plural samples prepared under the same conditions, 8 to 12 different areas (less than 5 μm **×** 5 μm) were scanned to observe 100 or more tethering spots at scanning speeds of 8 to 30 μm/s using ultrashort cantilevers (spring constant ~0.3 N/m, NanoWorld).

## Results and discussion

### Fabrication process of tethering nanoarray chip

**Fig 2:**
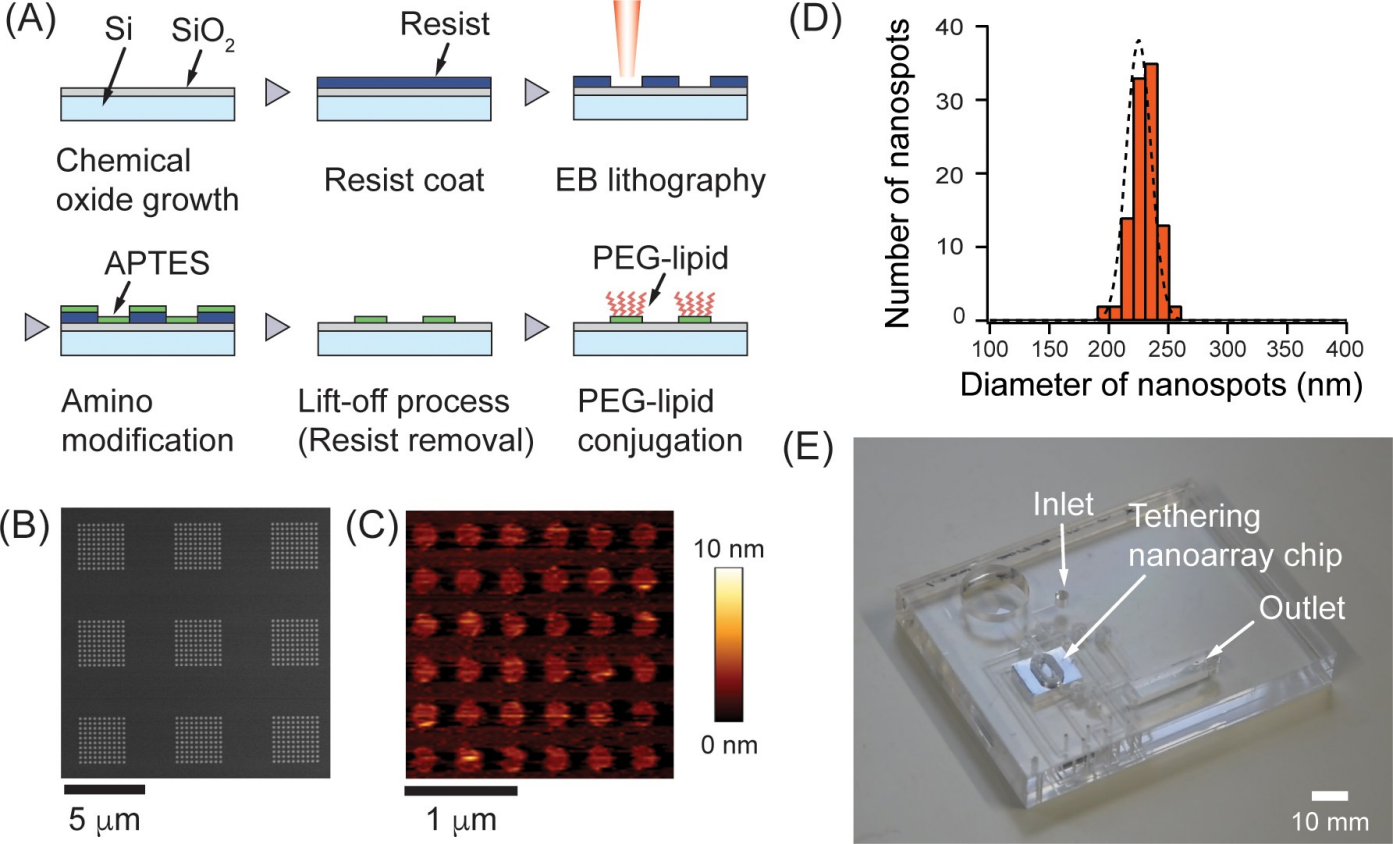
Hybrid nanofabrication process for forming an array of nanospots. (A) Schematic that depicts steps for fabricating PEG-lipid modified nanoarrays such as EB lithography, lift-off process, and selective chemical modification. (B) SEM image of EB resists film perforated with the 200-nm diameter nanoholes array. Bright spots correspond to delineated nanoholes, whereas dark regions correspond to the resist film. (C) Typical AFM image of the nanospots after the modification of both PEG-lipids and methoxy PEGs on amino modified spots. (D) Distribution of the nanospot diameters (*n* = 100). (E) Image of tethering nanoarray chip embedded in the microfluidic device.

We developed a process flow to fabricate the tethering nanoarray chip, as briefly summarized below and shown in Fig 2A. A Si substrate was cleaned by immersing it in sulfuric peroxide mixture (H_2_SO_4_:H_2_O_2_ = 3:1) at 200°C for 30 min. It was then rinsed in ultrapure water, leading to the formation of a chemical oxide layer on the top surface. A 40-nm thick ZEP520A resist was spin-coated on the substrate and exposed to an EB at an acceleration voltage of 50 kV and a dose of 75 μC/cm^2^. An array of nanoholes was delineated in the EB resist film after development using a ZED-N50 developer, followed by rinsing with ZMD-B. The substrate was subsequently treated with an aqueous solution of APTES for 1 h under optimized conditions to form a quasi-monolayer amino film that acts as a linker between the SiO_2_ surface and the PEG-lipids. Then, only the amino modified nanospots were left by a lift-off process using DMAC. Finally, the PEG-lipids were conjugated with amino groups (100 μM, 37 ºC) to create conjugate brushes within the nanospots. Methoxy PEGs were reacted with residual amino groups to suppress the nonspecific adsorption of proteins or lipoproteins coexisting in the EV solution [30, 43]. Details of the chemical modification process can be found in S1 Text and S1 Fig.

We examined fabrication steps, such as resist patterning, selective chemical modification, and formation of the nanospots, using various methods such as SEM, fluorescence labeling, and AFM. The SEM image in Fig 2B shows an array of the nanoholes with a mean diameter of 195 ± 6.8 nm (*n* = 40) fabricated in the EB resist film. The APTES molecules reacted with the Si surface in the fabricated nanoholes; however, the remaining EB resist film served as a mask and completely protected the other area. To investigate the effect of lift-off process on the activity of the modified molecules, the modified surface was fluorescently examined with amino groups and the amino group/PEG-lipid conjugates (see S1 Fig for detailed information). The patterns were selectively modified and prepared to react with the expected reactants. The AFM image in Fig 2C shows the fabricated PEG-lipid modified nanospots. Based on the histogram in Fig 2D, the nanospots had a mean diameter of 229 ± 11 nm (*n* = 100) and a corresponding coefficient of variation, *c*_*v*_, of 4.8%. Though the diameter of the fabricated nanospots exceeded 200 nm, PEG-lipid molecules located at the periphery of the spots may fall off and become attached to the substrate. In fact, ~90% of the nanospots had a height ranging from 2 to 3 nm, with a standard deviation of 0.7 nm. The height of the deformed EV is estimated from the height of the phospholipid bilayer (3–4 nm) to be 7.0 nm or greater. Thus, immobilized EVs can be recognized on these spots [44].

### AFM measurements of EVs on a tethering nanoarray chip

An EV suspension obtained from Sk-Br-3 or HEK293 cells was exposed to the tethering nanoarray, and the immobilized EVs were analyzed using AFM. After installing the nanoarray chip in the microfluidic device, 1 ml of the EV suspension (Sk-Br-3: 2.0 × 10^9^ particles/ml, HEK293: 1.5 × 10^10^ particles/ml, respectively) was applied to the chip surface at a flow rate of 7.5 ml/min and incubated for 24 h at room temperature, followed by a washout using PBS. We used a lower flow rate than in our previous report on immobilization of HEK239-derived EVs [35] to increase the immobilization rate because the total tethering area within the nanoarray chip is less than the non-nanopatterned surface. The nanoarray chip was then detached from the microfluidic device and observed using AFM in PBS. Subsequently, the chips were carefully processed to prevent the surface from drying before the AFM measurements. Details for the procedures, such as sample injection, EV immobilization, and AFM measurements, are provided in the supporting information (S1 Movie).

**Fig 3:**
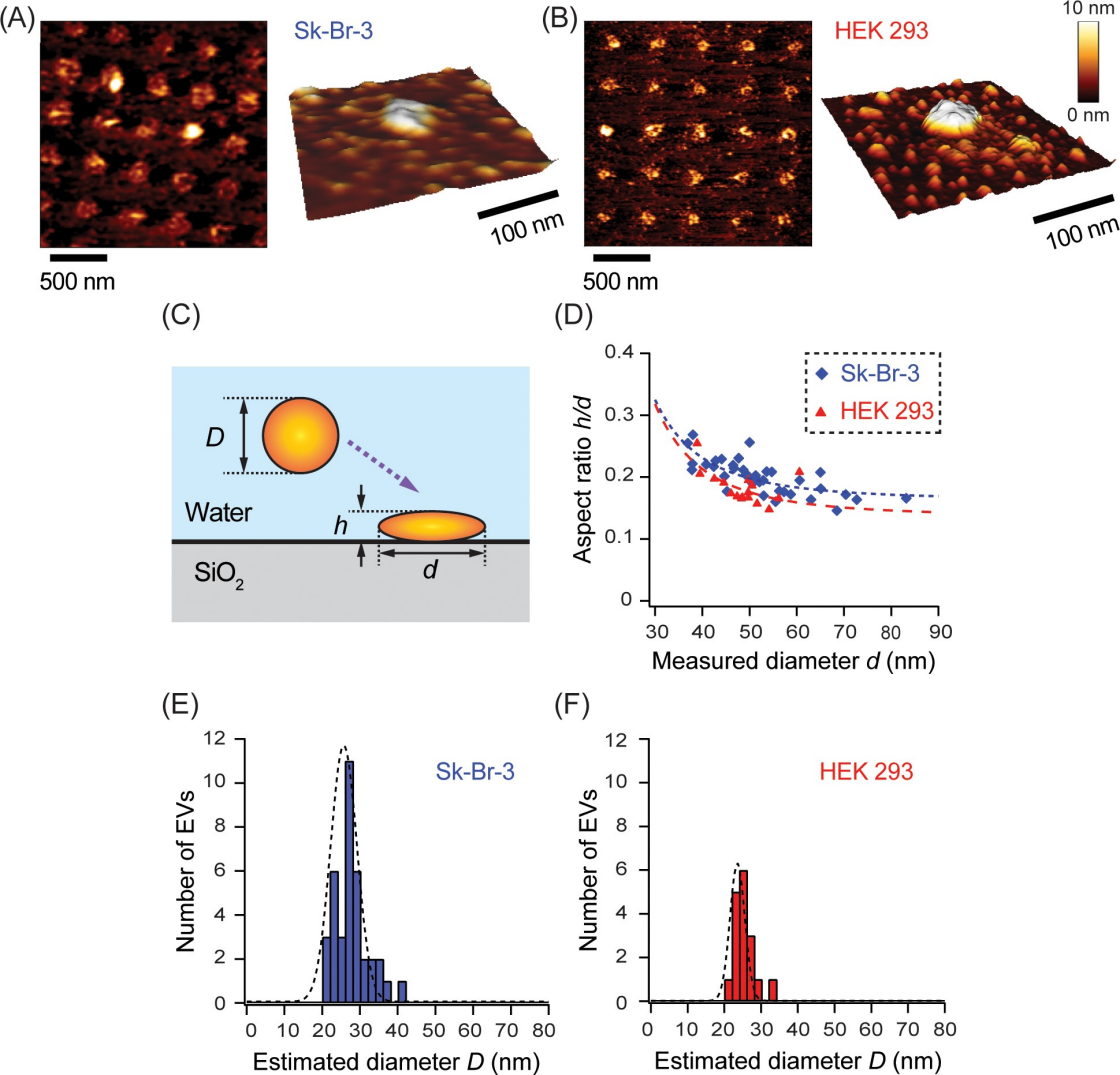
The immobilization of single EVs on the nanoarray chip. (A) and (B) AFM images of the nanoarray chip surface with tethered EVs and three-dimensional AFM images of an EV immobilized on a PEG-lipid modified nanospot. The EVs were (A) derived from Sk-Br-3 cells and purified by ultracentrifugation followed by density gradient fractionation; (B) derived from HEK293 cells and purified by ultracentrifugation. (C) Schematic showing dimensions of the EVs upon absorption. The diameter of the adsorbed EVs (*d*) is the mean the major and minor radii measured using AFM. Assuming no volume change by adsorption, the diameter of the corresponding EVs in the suspension (*D*) is estimated from *d* and *h*. (D) Relationship between the AR and *d* of EVs tethered on the nanospots. The curves (AR ~ *d*^−3^) were fitted using least square methods. Distribution of *D* of EVs derived from (E) Sk-Br-3 cells, (F) HEK293 cells.

As Fig 3A and 3B show, particles were separately immobilized on the tethering nanospots. A particle was regarded as an EV when the measured diameter and height exceeded 30 nm and 7 nm, respectively. About 20% of the nanospots were occupied by EVs in both cases, indicating the possibility that >10^5^ EVs were concurrently tethered to the nanoarray chip containing 10^6^ nanospots. Properly optimizing conditions such as flow rate enables shorter incubation times of 24 h and higher immobilization rates of 20% to achieve high-performance of the platform, as we demonstrated in previous study [35].

The adsorbed particles were not spherical as seen in the detail AFM images. They were observed as flattened like oblate ellipsoids, resembling the shape of exosomes derived from an oral cancer patient [17]. They are considered EVs due to the characteristic shape of lipid membrane vesicles. Thus, EVs tethered on the nanospots were deformed. The deformability was quantified by the aspect ratio (AR) defined as, AR = *h/d*, where the *d* denotes the mean diameter calculated from the major and minor radii and the *h* denotes the height of the deformed EV (Fig 3C). AR corresponding to EVs with a diameter of 35–85 nm derived from both Sk-Br-3 and HEK293 cell lines varied from 0.30 to 0.15, as shown in Fig 3D. In the size range of immobilized EVs in this study, the ARs decreased with an increase in *d* of the EVs measured for both cell lines, suggesting that larger EVs have greater deformability. Suppose that EVs with an estimated diameter in the liquid (*D*) are deformed by adsorption on the nanospot without a change in their volume and the resultant EV shape is an ellipsoid [45], then, *D* can be obtained using *d* and *h*. Based on this assumption, the AR is proportional to *d*^−3^. In this case, the calculated ARs were smaller than those obtained from the liposome model, in which the ARs ranged from 0.4 to 0.5 for EVs of equivalent size [36]. The result indicates the presence of a strong hydrophobic interaction between the PEG-lipids and the EV membrane. Hence, it represents the adhesive properties of the EV’s surface. Using a two-sample independent Student’s *t-*test at 95% confidence level, we observed a significant difference in the mean ARs of the tethered EVs between the originating cell lines (*p* < 0.05) (Sk-Br-3: AR = 0.20; HEK293: AR = 0.18). Since the cancer cell membranes have a different stiffness compared to normal cells, this preliminary attempt demonstrates that the deformability of EVs is correlated to their originating cells [46]. In the detailed images shown in Fig 3A and 3B, nanoparticles smaller than the EVs (< 20 nm) were observed at the periphery of the nanospots; the majority were considered low-density lipoproteins (LDL), which is difficult to be separated from EVs via density gradient ultracentrifugation [30, 43, 47, 48]. The small particles were in the shape of circular bulging and have an AR of about 0.3–0.6, which is considered morphologically distinguishable from EVs.

The mean *D* from the size of the immobilized Sk-Br-3 and HEK293 EVs was 27.7 ± 4.7 and 25.0 ± 2.6 nm, respectively, as shown in Fig 3E and 3F. The mean diameters of EVs derived from the Sk-Br-3 and HEK293 cells measured by applying the DLS method were 133 ± 60 and 167 ± 120 nm, respectively, as shown in S3 Fig. Comparing these values indicates that even among smaller EVs, such as exosomes (30–150 nm), relatively small EVs with a diameter of about 30 nm were selectively immobilized on the 200-nm diameter nanospots. Unlike the expectation at the time of design, larger tethering spots are required to immobilize EVs with a diameter of 100 nm under the current exposure condition.

We will now discuss the causes of size selectivity of the tethered EVs. There are two forces acting on an EV when it is attached to a tethering surface: a drag force derived from laminar flow and a resistance force derived from the hydrophobic interaction between the lipid bilayer and oleyl group at the end of the PEG-lipids. EVs generally tend to flow away before tethering when the drag force exceeds the resistance force. This behavior accounts for the immobilization of relatively small EVs on the nanospots because the drag force acting on a sphere with a diameter, *D* increases as the square of *D* increases [49]. In principle, the use of low-flow rates and strong hydrophobic groups at the end of PEG-lipids decrease the drag force and increase the resistance force, respectively. Further research investigating the correlations between the size of immobilized EVs and dominating factors, such as flow rate, type of probe molecules used and size of tethering spots, is beneficial for optimizing the performance of the platform.

Finally, we refer to the perspective of the platform developed in this work. By establishing a platform through individually immobilizing and evaluating EVs, our future work involves morphological feature analysis of individual EVs combined with machine learning methods and multivariate analysis. The nanoarray will be associated with spectroscopic measurements such as enzyme-linked immunosorbent assay (ELISA) [50], SERS [22, 51], and matrix-assisted laser desorption/ionization-time of flight (MALDI-TOF) mass spectroscopy [52] to identify molecular components. This platform is expected to offer new ways for multiplexed omics analysis of individual EVs, which are integrated with various measurements to address the challenges of heterogeneity in EV population, in addition to the clinical cancer diagnosis.

## Conclusions

We presented a new nanoarray platform that enables the immobilization of various EVs on surfaces for further analysis. The tethering nanoarray chips has a density of 5.0 × 10^5^ spot/mm^2^ and were achieved by applying a combination of microfluidic devices and nanopatterned PEG-lipid conjugates. We formed 10^6^ nanospots on a chip, where ~20% of the spots were simultaneously occupied by flattened EVs with relatively small diameters (*D* < 40 nm, in liquid). The deformability of the EVs, quantified by AR of the immobilized EVs, was greater than that obtained from the liposome model, thus demonstrating strong hydrophobic interactions between the PEG-lipids and the EV’s membranes. AR corresponding to EVs derived from both Sk-Br-3 and HEK293 cell lines varied from 0.30 to 0.15 with an increase in *d* on the surface, and a mean AR was 0.20 and 0.18, respectively, in the size range of immobilized EVs in this study. The characteristic deformabilities of EVs derived from different cell lines were morphologically detectable using the array platform. Thus, we showed that AR can be used as an indicator to evaluate the deformability of EVs, which is related to their adhesive and mechanical properties, using the EV nanoarray chip-based platform developed in this study.

## Supporting information

S1 Text Selective chemical modification. (DOCX)

S1 Movie EV analysis procedure on the tethering nanoarray-based platform. The movie shows a sequence of the procedure from fluid processing to EV analysis, featuring sample injection, EV immobilization under circulating flow, washout, uninstalling the chip, and analysis using AFM. To clarify the movement of the liquid, blue dyes were added to the solution displayed in the movie.
(MOV)

S1 Fig Modification procedure and fluorescence images of the modified surfaces. (A) Stepwise-chemical modification of the Si substrate to prepare its surface to capture EVs. Fluorescence images of (B) amino modified spots labeled with NHS-fluorescein (excitation, 488 nm; emission, 525–550 nm), (C) amino/PEG-lipids/methoxy PEG modified spots using rhodamine-DHPE (excitation, 561 nm; emission, 617–673 nm) to check reactivity of modified molecules. The contrast in the images in S1 Fig (B, C) is enhanced to show the clear edge of the nanospots (200 nm in diameter, 1200 nm in pitch).
(TIF)

S2 Fig Detection of membrane proteins. Tetraspanins on the membranes of EVs derived from Sk-Br-3 cell lines were detected by Western blotting. Exosome marker proteins CD9, CD63, CD81 and Hsp70 existed on the lane of relevant fractions (6–9). The amount of total proteins colored with Thermo Micro BCA Protein Assay Kit (Thermo Fisher Scientific) were quantified using NanoDrop (ND-1000, Thermo Fisher Scientific), and prepare the sample for 0.3 μg/lane. Samples were resuspended in 4×Laemmli buffer (#1610747, Bio-Rad) and heated for 5 min at 95°C. Electrophoresis (50 mA, 75 min) and transfer were performed using an automatic transfer electrophoresis apparatus DIRECT BLOT (BM-80, Sharp Life Science) using a dedicated SDS-PAGE polyacrylamide gel (10%, BM-810012) and Immbilon-P membranes (polyvinylidene difluoride; pore size, 0.45 μm; Merk Millipore). Western blotting was performed using iBind Flex Western Device and iBind Flex solution for blocking (Thermo Fisher Scientific). Primary antibodies (1 mg/ml, Cosmo Bio), anti-CD9 (#SHI-EXO-MO1, ×5000 dilution), anti-CD63 (#SHI-EXO-MO2, ×500 dilution) and anti-CD81 (#SHI-EXO-MO3, ×5000 dilution), and were diluted with iBind Flex solution. Anti-Hsp70 (#EXOAB-Hsp70A-1, 0.25 mg/ml) was obtained from System Biosciences and diluted 500 times with iBind Flex solution. ECL peroxidase labelled anti-mouse antibody (#NA931VS, GE Healthcare) diluted 1000 times with iBind Flex solution was used as a secondary antibody. Amersham ECL select western blotting detection reagent (#RPN2235) and Amarsham Imager 600 (GE Healthcare) were used for detection.
(TIF)

S3 Fig Size distribution of EVs in suspension. The size distribution of EVs secreted from (A) Sk-Br-3 cell lines and (B) HEK293 cell lines. The size distributions were measured using NTA to ensure selectivity of the EV size in the tethering process. The mean diameters of the histograms (A) and (B) were 133 ± 60 and 167 ± 120 nm (*n* = 900 and 240), respectively.
(TIF)
