# Supplementary material for: Extracellular vesicles nanoarray technology: Immobilization of individual extracellular vesicles on nanopatterned polyethylene glycol-lipid conjugate brushes
Source: PLoS One. 2019 Oct 24;14(10):e0224091. doi: 10.1371/journal.pone.0224091 (PMC6812765; doi:10.1371/journal.pone.0224091)
Supplement: S1 Text — (DOCX) [file pone.0224091.s001.docx]

S1 Text. Selective chemical modification.

The procedure for selective chemical modification (corresponding to the lower row of Fig 2A) is illustrated in S2 Fig A. APTES is used as a linker between PEG-lipids and SiO_2_ surface. The reactivity of the surface amino-group obtained from APTES is a key when grafting PEG-lipids. However, the reactivity may be reduced during the lift-off process performed in an organic solvent. To evaluate the effect of the lift-off process on the reactivity, amino modified nanospots were labeled with *N*-hydroxysuccinimide (NHS)-fluorescein (Thermo Scientific) and observed using a spinning disk confocal super resolution microscope (SpinSR10, Olympus) with an oil immersion objective lens (UPLAPO OHR 60x), as shown in S2 Fig B. We compared the fluorescence intensities obtained before and after removing the resist and found that about 80% of the amino groups were active after the lift-off, indicating that the lift-off had little effect on the reactivity of the amino group. This result confirms the possibility of further modifying the surface amino group with NHS-terminated PEG-lipid. S2 Fig C illustrates the fluorescent image of the PEG-lipid and methoxy PEG modified nanospots labeled with Lissamine™ rhodamine B 1,2-dihexadecanoyl-*sn*-glycero-3-phosphoethanolamine (Rhodamine-DHPE, Thermo Scientific). A clear contrast was observed in the fluorescence intensity, confirming that the modified PEG lipid molecules can react with the lipid-containing material. The process can be applied to micro- and nano-patterning with relatively small loss in the reactivity of the modified molecules.
