# Supplementary figures and images for: Extracellular vesicles nanoarray technology: Immobilization of individual extracellular vesicles on nanopatterned polyethylene glycol-lipid conjugate brushes
Source: PLoS One. 2019 Oct 24;14(10):e0224091. doi: 10.1371/journal.pone.0224091 (PMC6812765; doi:10.1371/journal.pone.0224091)

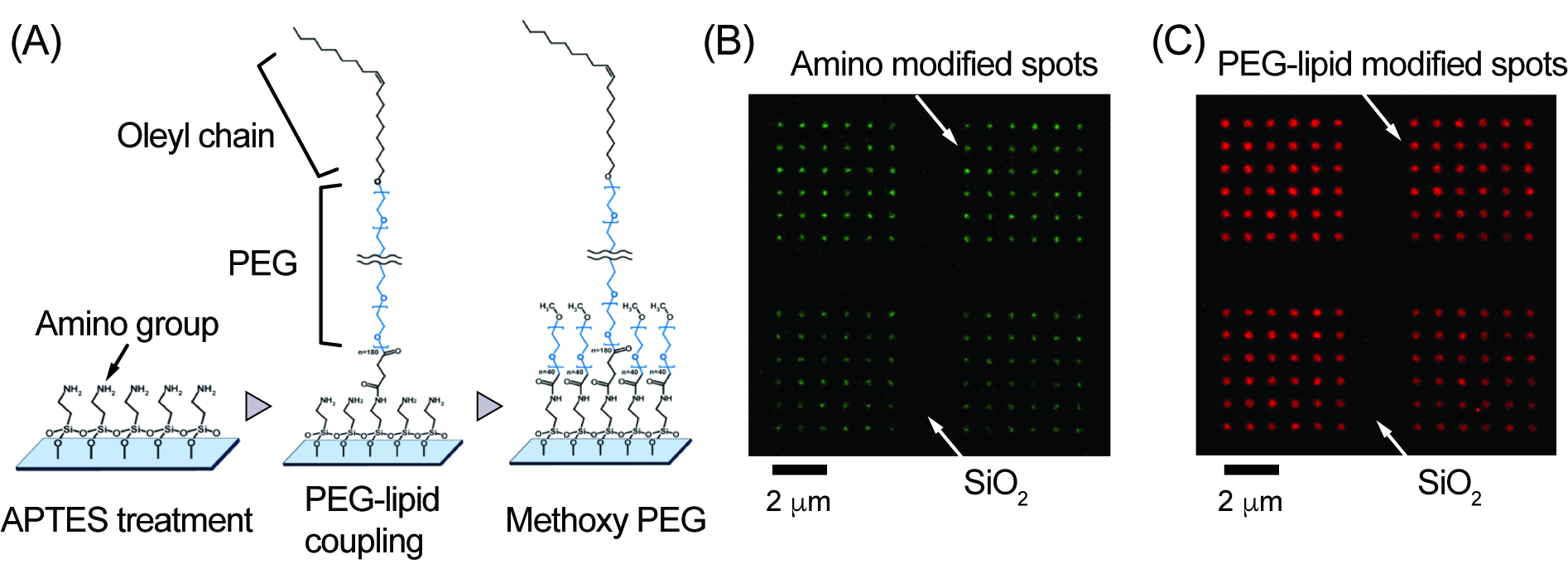

Supplement: S1 Fig — (A) Stepwise-chemical modification of the Si substrate to prepare its surface to capture EVs. Fluorescence images of (B) amino modified spots labeled with NHS-fluorescein (excitation, 488 nm; emission, 525–550 nm), (C) amino/PEG-lipids/methoxy PEG modified spots using rhodamine-DHPE (excitation, 561 nm; emission, 617–673 nm) to check reactivity of modified molecules. The contrast in the images in S1 Fig (B, C) is enhanced to show the clear edge of the nanospots (200 nm in diameter, 1200 nm in pitch). (TIF) [file pone.0224091.s003.tif]

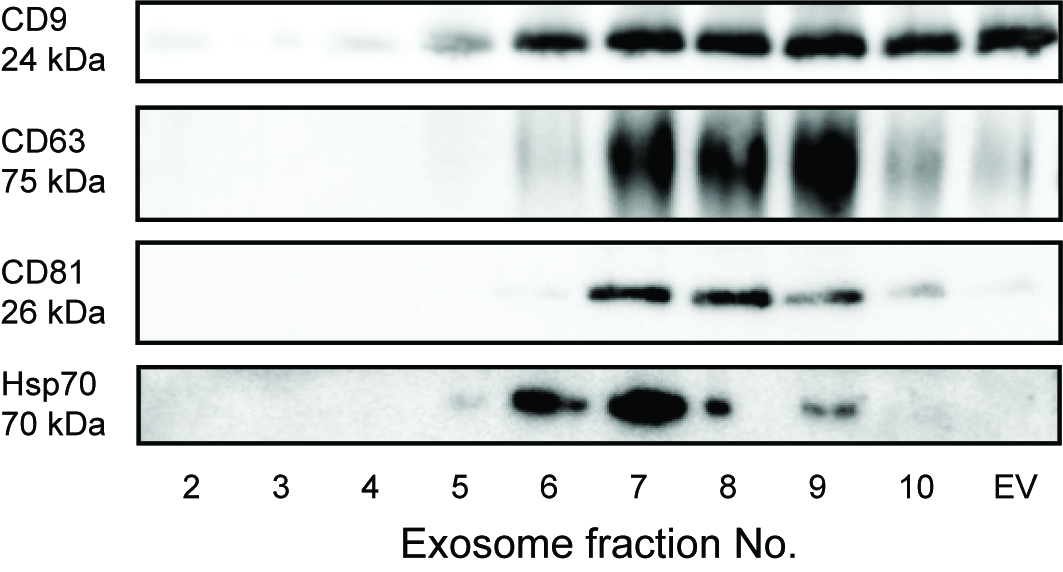

Supplement: S2 Fig — Tetraspanins on the membranes of EVs derived from Sk-Br-3 cell lines were detected by Western blotting. Exosome marker proteins CD9, CD63, CD81 and Hsp70 existed on the lane of relevant fractions (6–9). The amount of total proteins colored with Thermo Micro BCA Protein Assay Kit (Thermo Fisher Scientific) were quantified using NanoDrop (ND-1000, Thermo Fisher Scientific), and prepare the sample for 0.3 μg/lane. Samples were resuspended in 4×Laemmli buffer (#1610747, Bio-Rad) and heated for 5 min at 95°C. Electrophoresis (50 mA, 75 min) and transfer were performed using an automatic transfer electrophoresis apparatus DIRECT BLOT (BM-80, Sharp Life Science) using a dedicated SDS-PAGE polyacrylamide gel (10%, BM-810012) and Immbilon-P membranes (polyvinylidene difluoride; pore size, 0.45 μm; Merk Millipore). Western blotting was performed using iBind Flex Western Device and iBind Flex solution for blocking (Thermo Fisher Scientific). Primary antibodies (1 mg/ml, Cosmo Bio), anti-CD9 (#SHI-EXO-MO1, ×5000 dilution), anti-CD63 (#SHI-EXO-MO2, ×500 dilution) and anti-CD81 (#SHI-EXO-MO3, ×5000 dilution), and were diluted with iBind Flex solution. Anti-Hsp70 (#EXOAB-Hsp70A-1, 0.25 mg/ml) was obtained from System Biosciences and diluted 500 times with iBind Flex solution. ECL peroxidase labelled anti-mouse antibody (#NA931VS, GE Healthcare) diluted 1000 times with iBind Flex solution was used as a secondary antibody. Amersham ECL select western blotting detection reagent (#RPN2235) and Amarsham Imager 600 (GE Healthcare) were used for detection. (TIF) [file pone.0224091.s004.tif]

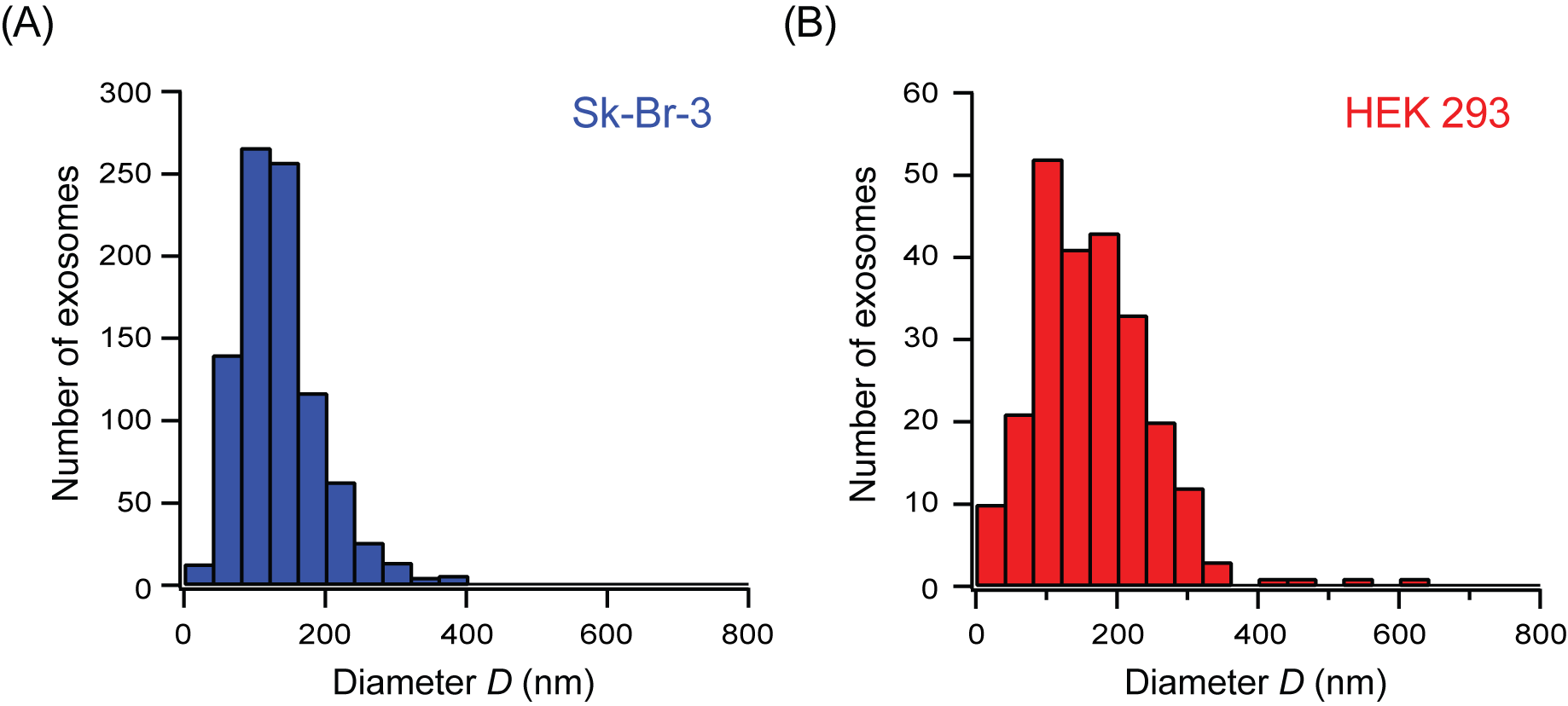

Supplement: S3 Fig — The size distribution of EVs secreted from (A) Sk-Br-3 cell lines and (B) HEK293 cell lines. The size distributions were measured using NTA to ensure selectivity of the EV size in the tethering process. The mean diameters of the histograms (A) and (B) were 133 ± 60 and 167 ± 120 nm (n = 900 and 240), respectively. (TIF) [file pone.0224091.s005.tif]
